# GLYCEMIC BEHAVIOR IN 48 HOURS POSTOPERATIVE PERIOD OF PATIENTS WITH TYPE 2 DIABETES MELLITUS AND NON DIABETIC SUBMITTED TO BARIATRIC SURGERY

**DOI:** 10.1590/S0102-6720201500S100009

**Published:** 2015-12

**Authors:** Lucas Freitas de OLIVEIRA, Caroline Gewehr TISOTT, Diego Machado SILVANO, Camila Mafalda Mouta CAMPOS, Ricardo Reis do NASCIMENTO

**Affiliations:** 1 Santa Catarina South University; 2 Nossa Senhora da Conceição Hospital, Tubarão, SC, Brazil

**Keywords:** Bariatric surgery, Metabolic surgery, Type 2 diabetes mellitus

## Abstract

Although there is no indication for surgery taking only into account the glycemic condition, results have shown that benefits can be obtained in glycemic control with bariatric surgery.

**Aim:**

: To compare the glycemic behavior among type 2 diabetic and non-diabetic patients within 48 h after bariatric surgery, and clarify whether there is a reduction in blood glucose level in obese patients with diabetes before the loss of weight excess.

**Methods:**

: Descriptive epidemiological study with prospective cohort design with 31 obese patients undergoing Roux-en-Y gastric bypass and sleeve gastrectomy. The patients were controlled with hemoglucotests in different periods of time: preoperative, postoperative and each 6 h after surgery for 48 h.

**Results:**

: Average ambulatory blood glucose in diabetics was 120.7±2.9 mg/dl vs 91.8±13.9 mg/dl in the nondiabetic. After 48 h there was decrease in diabetics to 100.0±17.0 mg/dl (p=0.003), while the non-diabetic group did not change significantly (102.7±25.4 mg/dl; p=0.097). There were no differences between the surgical techniques. There were no death.

**Conclusions:**

: Diabetic patients significantly reduced blood glucose after surgery regardless of the use of exogenous insulin or oral hypoglycemic agents.

## Abstract (pt)

**Racional:**

Apesar de não haver indicação cirúrgica levando-se exclusivamente em consideração apenas o estado glicêmico, resultados já revelaram que pode ser obtido benefício no controle da glicemia com operação bariátrica.

**Objetivos:**

: Comparar o comportamento glicêmico dos pacientes diabéticos tipo 2 ou não nas 48 h após cirurgia bariátrica, e esclarecer se há redução nos níveis glicêmicos de obesos com diabete antes da perda de excesso de peso.

**Métodos:**

: Realizou-se estudo epidemiológico descritivo com delineamento de coorte prospectiva de 31 pacientes obesos submetidos ao bypass gástrico em Y-de-Roux e gastrectomia vertical. Acompanhou-se o hemoglicoteste nos momentos pré, trans, pós-operatório e a cada 6 h após a operação durante 48 h.

**Resultados:**

: A glicemia ambulatorial média nos diabéticos era 120,7±2,9 mg/dl vs 91,8±13,9 mg/dl nos não diabéticos. Após 48 h houve queda nos diabéticos para 100,0±17,0 mg/dl (p=0,003), enquanto o grupo não diabético não se modificou de forma significante (102,7±25,4 mg/dl; p=0,097). Não houve diferenças quanto às técnicas cirúrgicas empregadas. Não houve óbito.

**Conclusões:**

: Os pacientes diabéticos reduziram significativamente a glicemia após a operação independentemente do uso de insulina exógena ou drogas hipoglicemiantes orais.

## INTRODUCTION

Nowadays, due to the nutritional transition, obesity and type 2 diabetes mellitus (T2DM) have been considered an epidemic disease. OMS data from 1980 through 2008 show that the prevalence of obesity has more than doubled within this period^27^. In the year 2011 in Brazil there were studies from the data of Risk and Protective Factors Surveillance for Chronic Diseases by Telephone Interviews (VIGITEL) of the Ministry of Health, and it was estimated that 48.5% of the adult population was in excess of range weight (BMI>25 kg/m²) and in the same population, 15.8% were obese^18^. 

With regard to T2DM, in the year 2009 it was estimated a worldwide prevalence of 6.4%, and projections for 2030 that show the possible increase of 69% in the number of diabetic adults in countries in developement^24^. Today, these disorders that were previously restricted to older adults are now increasingly focused on children and adolescents^28^.

In 2012, the United States of America estimated that the direct cost with diabetic patients was about 245 billion dollars, representing an increase of expenditures on the order of 41% when comparing to the last review in 2007^2^. When analyzing what both these conditions cause in human homeostasis^28^, a plan is to be generated for a future scenario where young ones and adults that were disabled permanently by sequelae and could have been prevented from secondary diseases to metabolic disorders, generating great economic outlay for states and health systems.

Regarding the forms of treatment for obesity, meta-analyzes were performed comparing surgical approach with isolated clinical treatment, showing superiority of invasive option both in terms of weight loss as improvement of comorbidities^8^^,^ 17.

As for the T2DM, although no surgical indications taking into consideration for only the glycemic status of the patient, the results obtained with patients who had precise indications for bariatric surgery revealed that the benefit could be obtained in glucose postoperative control. Schauer et al.^22 ^compared the standard drug treatment intensively for glycemic control to the association of hypoglycemic drug therapy with gastric bypass Roux-en-Y (RYGB) and found better glycemic control rates after 12 months in the second group (HbA1c 7. 5±1.8% vs 6.4±0.9%, p<0.001). In this same study, the loss of weight was higher in the group associated to surgical option to the clinic (-9.4±9.0 kg vs -5.4±8.0 kg; p<0.001). There were no death or lethal complications while these studies were performed.

Furthermore, loss of excess weight seems to give positive effect in preventing the development of metabolic disorders related to obesity. Carlsson et al.^6^ compared the incidence of T2DM between two non-diabetic groups submitted and also not submitted to bariatric surgery. With follow-up of 15 years, it was found that the incidence was respectively 6.8 and 28.4 cases per thousand patients a year, and operative mortality was 0.2%. It was concluded that the operation for obesity was superior to clinical treatment to prevent new cases of T2DM in obese. Therefore, it is undeniable the efficacy that bariatric surgery has in the handling of patients who are overweight and glucose metabolism alterations when conventional therapeutic measures were ineffective.

However, what was not known was that the benefit in glycemic profile obtained after the operation could already be evident even before the loss of excess weight^21^. We try to explain this effect prepared by association of factors that lead to increased peripheral insulin sensitivity and improved function of β-pancreatic cells^9^^,^ 20.

On the other hand, the immune-endocrine-metabolic insult inherent in the surgical procedure leads to slant catabolic changes on the exposed organism. Among the final products of this intricate and complex network of response to aggression, the alteration in blood glucose draws attention in the specific case of bariatric surgery in diabetics as it is subject to questioning whether the dysfunction of pancreatic beta cells prior to surgical trauma can significantly change this answer.

Therefore, it is inserted in this context that the present study aims to compare the glycemic behavior between diabetic and non-diabetic patients within 48 hours following the surgery, and clarify whether there is a reduction in glucose levels of obese patients with T2DM prior to post-surgical loss of excess weight.

## METHODS

This study was approved by the Ethics Committee in UNISUL by the number of 24697813.2.0000.5369 protocol in respect to Resolution 466 of 2012 of the National Health Council.

Prospective cohort study was carried out with a sample of 31 patients submitted to RYGB and sleeve gastrectomy (GV).

Were excluded the patients who used insulin prior to treatment, GLP-1 analogues medications, DPP-IV inhibitors, those who required admission to the intensive care unit postoperatively and those that did not contain enough data in the chart.

The following preoperative laboratory parameters were used: high density lipoprotein, low density lipoprotein, triglycerides and fasting glucose by colorimetric enzymatic method. In addition, was took into consideration the calculation of body mass index (BMI), as recommended by OMS^26^. Was evaluated the blood glucose at the following times: induction, trans-operative, immediate postoperative, 6 h after the end of the operation, every 6 h from first to second day after surgery.

The collected data were entered into the Epi Info 3.5.4 program and the statistical analysis was made with their assistance. It is considered statistically significant p​<0.05.

## RESULTS

The study included 31 participants, and of these 77.4% were women. Of the total, 32.3% had TDM2. With regard to the type of operation, 80.6% were submitted to RYGB. On average, the BMI group submitted to VG was 47.5±9.8 kg/m². Already, the group that had RYGB, the average was 41.5±5.4 kg/m², and this difference was statistically significant (p=0.049). The average age was 38.2±10.4 years (20-65) and the age of each group was 42.3±18.8 years in submitted to VG and 37.4±8.9 years in the RYGB (p=0.312). The characteristics of the patients are shown in Table 1.

**TABLE 1:** - Characteristic of groups (n=31)

	**VG**	**RYGB**	**Total**			
	**N**	**%**	**n**	**%**	**n**	**%**
Gender						
Male	2	28.6%	5	71.4%	7	100%
Female	4	16.7%	20	83.3%	24	100%
TDM2						
Yes	3	30%	7	70%	10	100%
No	3	14.3%	18	85.7%	21	100%

VG=vertical gastrectomy; RYGB=Roux-en-Y gastric bypass; TDM2=diabetes mellitus type 2

The blood glucose outpatient preoperative average of diabetics fasting was 120.7±2.9 mg/dl vs 91.8±13.9 mg/dl in the non diabetic (p=0.000).

As for the control of blood glucose immediately before surgery, those who did GV showed better rates compared to undergoing RYGB. In trans periods and immediate postoperative period there were no statistic significant differences between surgical techniques (Table 2).

**TABLE 2:** - Glycemia comparison between the two surgical groups (n=31)

	**VG**	**RYGB**	**p**
Capillary blood glucose average preoperative	90.8±11.6 mg/dl	120.4±33.7 mg/dl	0.045
Capillary blood glucose average intraoperative	139.1±27.3 mg/dl	139.3±25.7 mg/dl	0.987
Capillary blood glucose average immediate postoperative	158.6±35.5 mg/dl	154.9±33.8 mg/dl	0.813

VG=vertical gastrectomy; RYGB=Roux-en-Y gastric bypass

During the operation, the average blood glucose of diabetic patients was 143.8±31.0 mg/dl. In those without diabetes, the figure was 137.1±23.0 mg/dl (p=0.510). Immediately after surgery, there was significant statistical difference between the hemoglicotest groups (180.3±35.2 mg/dl vs 143.9±26.2 mg/dl; p=0.003).

Regarding the values of blood glucose in the first two days after surgery, the diabetic group submitted to VG had an average of 116.3±20.7 mg/dl. Those whose RYGB the average value was 124.2±25.3 mg/dl (p=0.652). 

Between the two surgical groups of individuals yes/no diabetes, the average was respectively 110.3±11.5 mg/dL and 122.4±21.2 mg/dl (p=0.357). Using linear regression analysis the association attempts to metabolic profile variables with mean blood glucose range around there were no statistic significant associations (Table 3).

**TABLE 3:** - Linear regression relating mean postoperative hemoglucotest and preoperative metabolic variables (n=31)

**Variable**	**Coefficient**	**Std error**	**F-Test**	**p**
Total cholesterol	-0.122	0.406	0.0903	0.766
HDL	-0.194	0.408	0.2261	0.638
LDL	0.138	0.346	0.1596	0.692
Triglycerides	-0.026	0.088	0.0855	0.772

In evaluating the relationship between BMI and postoperative average capillary blood glucose, the linear correlation coefficient of Pearson was 0.62 (p=0.248).

Table 4 explains the glycemic variation in the period studied between diabetic and non-diabetic patients.

**TABLE 4:** - Glycemic behavior during the first 48 h after bariatric surgery (n=31)

	**Diabetics**	**Non-diabetics**	
HGT 6 h PO	130.7±23.8 mg/dl	137.3±29.8 mg/dl	0.547
HGT 12 h PO	129.5±28.2 mg/dl	141.6±39.9 mg/dl	0.395
HGT 18 h PO	139.0±38.7 mg/dl	130.1±33.5 mg/dl	0.515
HGT 24 h PO	129.3±35.4 mg/dl	121.9±30.1 mg/dl	0.551
HGT 30 h PO	117.2±22.2 mg/dl	113.8±28.9 mg/dl	0.746
HGT 36 h PO	117.5±32.1 mg/dl	112.0±24.2 mg/dl	0.602
HGT 42 h PO	111.4±27.0 mg/dl	105.9±24.2 mg/dl	0.573
HGT 48 h PO	100.0±17,0 mg/dl	102.7±25.4 mg/dl	0.758

HGT=hemoglucotest; PO = postoperative

In patients with TDM2, there was a reduction with statistical significance of ambulatory blood glucose before surgery compared to hemoglucotest held at 48 h postoperatively (Table 4).

**TABLE 5:** - Paired test samples (n=31)

**TDM2**	**Average**	**p**	
	**Ambulatory blood glucose**	**HGT48 h postoperatively**	
Yes	120.7±12.9 mg/dl	100.0±17.0 mg/dl	0.003
No	91.8±13.9 mg/dl	102.7±25.4	0.097

HGT=hemoglucotest

In the postoperative follow-up, both groups showed significant decrease of glycemia (Table 6).

**TABLE 6:** - Paired test samples (n=31)

**TDM2**	**Average**	**p**	
	**HGT 6 h postoperatively**	**HGT 48 h postoperatively**	
Yes	130.7±23.8 mg/dl	100.0±29.8 mg/dl	0.002
No	137.2±29.8 mg/dl	102.7±25.4 mg/dl	0.000

HGT=hemoglucotest

There were no deaths nor any life threatening complication to patients.

## DISCUSSION

In this study they predominated patients undergoing RYGB (80.6%). On a global survey conducted in 2011, of the 340,768 bariatric procedures performed, 46,6% were bypassed. In contrast, in the same year, Brazilian surgeons used the same technique in 70% of its procedeures^11^. Therefore, although the evidence is present here they comply with national status, both are different from international. This may be explained by different assumptions; however, the technique of choice is headed by the patient's opinion and based on the experience of the surgical team, underlying medical condition of the patient, realistic goals and objectives of the surgical outcome.

Analyzing and comparing the BMI of both groups of this study, a statistic significant difference is noticed, and the VG index was higher on patients (47.5±9.8 kg/m² vs 41.5±5.4 kg/m²; p=0.049). This may have been the result of less surgical time, not making anastomoses with its inherent risks and its technical feasibility^15^ when compared to other surgical option in this group of higher grading obesity, which naturally carries greater difficulties compared to less obese. Also, it can become an attractive option to allow initial weight loss in morbidly obese patients, making it reach lower surgical risks, allowing it to finalize its operation in the second half with intestinal bypass.

Regarding the lipid profile this study sought influences in glycemic control after the operation. However, this association was not verified and preoperative dyslipidemia does not influence negatively.

Another point of this study was to question whether preoperative BMI could influence postoperative glycemic behavior. Thus, it could not find statistical correlation between these variables (index of Pearson 0.62; p=0.248); therefore the glycemic decrease observed was not different between individuals depending on the degree of obesity. In a study within 24 months of follow-up, Teixeira et al.^25^ also noted that the preoperative BMI did not influence the glycemic improvement after the operation. Thus, this index does not seem to predict worse metabolic response to the procedure.

With regards to the adjustments in blood glucose in the moments before, during and after surgery, the only difference here witnessed was spotted at the time immediately after the procedure. Diabetic patients had hemoglucotest average 180.3±35.2 mg/dl, whereas those non-diabetics had the average of 143.9±26.2 mg/dl (p=0.003). Several factors influence the blood levels of glucose in surgical patients^12^: response endocrine-metabolic and immunological inherent in the procedure, the presence of TDM2, anesthesia, preoperative anxiety and any acute complications. The neuroendocrine response begins the days preceding the surgery, upon installation anxiety disorders, culminating in catecholaminergic discharge. The anesthesia and tracheal intubation still release more noradrenaline and adrenaline. The surgical incision to trigger immune activation, cytokines and interleukins which unloads the work on spinal cord via the central level so as bloodstream trigger the hypothalamic-pituitary-adrenal axis, amplifying hormone discharge already installed. Therefore, when creating internal environment dominated by counter-regulatory hormones insulin, such as adrenocorticotropic hormone, cortisol, glucagon, growth hormone, epinephrine, norepinephrine, and several cytokines, it is nurtured the rise of blood glucose levels. The importance of this lies in the fact that hyperglycemic patients have difficulty chemotaxis and phagocytosis of leukocytes, reduction in the expression of adhesion molecules, decrease in the complement system functionality, reduced nitric oxide synthesis and consequent injury to vasodilation. All these changes may predispose to increased inflammation, exacerbated vulnerability to infections and organ dysfunction^16^. While it is clear that hyperglycemia can be harmful to the body in the postoperative time, they lack scientific evidence to demonstrate that strict glycemic control, aiming values between 80 and 110 mg/dl, should be used routinely in all. Until the subgroups of patients who may get better benefits and strategies are defined, the American Society of Anesthesiologists recommends that blood glucose levels are kept below 150 mg/dl^16^. Therefore, it is in the diabetic subgroup that appears to be most needed attention and care.

The central data detailed in this paper was that the preoperative outpatient glycemic control in diabetic patients was worse compared to non-diabetic patients (120.7±2.9 mg/dl vs 91.8±13.9 mg/dl, p=0.000). But during the 48 h to immediate operation were not caught significant differences between groups, and the choice of surgical technique did not influence the glycemic postoperative course. Another important point was the fall of the finding in blood glucose in the diabetic group already in sequential hours of operation compared to its outpatient values ​​(120.7±2.9 mg/dl vs 100.0±17.0 mg/dl, p=0.003). Furthermore, despite the greater hyperglycemic peak in response to surgical trauma, glycemic behavior of diabetics began to resemble the non-ill patients, as evidenced by the evolution glycemic in Tables 5 and 6. Therefore, after the procedure, the groups are similar if blood glucose is in the question, and it was found well before the loss of excess weight, which is the proposed procedure. Derive from this observation the chances crediting part of improving glucose to postsurgical functional modifications.

The diverse functional nature of changes in the gastrointestinal tract made after the surgery has been studied, but has been the focus enteric hormonal response to postoperative anatomical changes. In addition to the volumetric restriction of gastric chamber mechanically determine earlier feeling of satiety, several studies point out that the phenomenon of anorexia induced by bariatric surgery appears to be originating also from hormonal changes, resulting in reducing the orexigenic hormone ghrelin secretion by cells located at the gastric fundus such as by increasing circulating levels of hormones such as glucagon-like peptide-1 (GLP-1) and peptide YY (PYY), secreted by the L-type cells located in the distal portions of the small intestine, which act in the hypothalamic centers promote feeling of anorexia, leading the individual to satiety and decrease in caloric output^3^^,^ 10 ^,^ 13 ^,^ 14 ^,^ 19. The generated state leads to weight loss and indirect improvement in insulin sensitivity.

In the generated modulation of appetite regulatory centers in the central nervous system, enteric incretinic hormones have also been related to changes in the postprandial insulin response. Alves et al.^1^observed that 45 days after RYGB there is statistic significant reduction of insulin resistance (p=0.021). On the path of understanding metabolic changes caused by bariatric surgery, Dirksen et al.^9^recently elucidated some of the mechanisms responsible for the post-bariatric glycemic improvement. Before the loss of excess weight in providing decrease peripheral insulin sensitivity by reducing adipose tissue, few changes are generated in a matter of days to weeks. The liver tissue becomes less resistant to insulin action, appearing to be due mainly to the energy restriction. Also, phenomenon of gradual recovery of the function of pancreatic beta-cells occurs, making more physiological insulin secretion, thus bringing the euglycemic state to the patient. Another change observed was that of gut hormones. In addition to the role in inducing pattern anorectic mentioned above, GLP-1 enhances the postprandial insulin response. The mechanism leading to this modification is explained by early exposure of more distal segments of the small intestine gastroenterostomy provided by the Roux-en-Y and the increased intestinal transit rate after GV, causing food quickly reach the distal small. In these places, they are arranged the L cells responsible to secrete this peptide. Thus, it leads to the state of exaggerated secretion of GLP-1 which culminates in greater betapancreatic stimulation. And as the results obtained by Le Roux et al.^15^, this hormone picture starts to appear as early as 48 h postoperatively.

In the case of GV primary responsibility currently identified to explain the glycemic improvement is the hormone ghrelin, produced mainly by oxyntic cells located in the gastric pit. By stimulating neurons in hypothalamic arcuate nucleus, that substance stimulates food intake. With its withdrawal, possibly, lower food output will be generated, which may explain the improvement of glycemic profile. Blaire et al.^4^ conducted systematic review of the literature on the subject and found that there was a reduction of serum ghrelin 698.4±312.4 pg/ml to 414.1±226.3 pg/ml (p=0.000) after the operation. It is therefore considerable reduction nurtured by this, but more studies are needed to clarify the exact relationship of this observation.

Although the caloric restriction contributes to the reduction of blood glucose in immediate evaluation after the operation, Evans et al.^10^ demonstrated that post-surgical incretinic response is so regardless of this. In addition, keeping patient in highly restricted diet becomes unviable in the long term, while getting enteric response through the operation provides opportunities to achieve benefits without basing its power on restricted diet.

One of the difficulties encountered in this study was the fact that the articles published in national and international literature stuck only in longer postoperative evaluations, such as those made with 30 days or more. Therefore, they lacked data from different centers for comparison in relation to 48 h postoperative. Another limitation was the fact that assessment did not extend in the months following the operation, so as to build on the glycemic comparison between diabetics and non-diabetics. Thirdly, the measures which quantify the peripheral insulin resistance as the model for evaluation of HOMA-IR homeostasis, could not be assessed due to non biochemical measurement variables necessary for calculation for each patient that would be operated. Despite good glycemic response, the diabetic groups was heterogeneous; however, this study did not set out to highlight the differences that have led to behavioral discrepancy of these individuals and other research is needed to characterize more detail in this aspect. Furthermore, due to the small sample it was not possible to demonstrate statistic significant differences that would be found with larger samples.

Thus, the action spectrum of bariatric surgery should not be understood only from the perspective of loss of excess weight generated, but rather as wide action procedure, characterized modern metabolic, as initially proposed by Bunchwald^5^ who used the term to describe anatomical modifications surgically induced generating reduction or disappearance of updated functions that culminate in disease; or also functional changes in the opposite direction, offsetting partially or in full the function originally changed. In a review on the topic, Zeve et al.^29^ points out what has already been discussed: the need for parameters that identify patients with the best risk/benefit in the indication of surgery, not based only on BMI stratum. Thus the first hypothesis for the exact understanding of the mechanisms involved in optimizing the intermediary metabolism of carbohydrates after the anatomical changes proposed by bariatric surgery have already been made and can explain the results found in this study.

## CONCLUSION

Diabetic patients have significantly reduced blood glucose after the operation regardless of the use of exogenous insulin or oral hypoglycemic drugs.
